# Liver Transcriptome Changes of Hyla Rabbit in Response to Chronic Heat Stress

**DOI:** 10.3390/ani9121141

**Published:** 2019-12-13

**Authors:** Zhou-Lin Wu, Xue Yang, Shi-Yi Chen, Fei-Long Deng, Xian-Bo Jia, Shen-Qiang Hu, Jie Wang, Song-Jia Lai

**Affiliations:** 1 Farm Animal Genetic Resources Exploration and Innovation Key Laboratory of Sichuan Province, Sichuan Agricultural University, Chengdu 611130, China; wu9121@126.com (Z.-L.W.); chensysau@163.com (S.-Y.C.); fdeng@uark.edu (F.-L.D.); jaxb369@sicau.edu.cn (X.-B.J.); sqhu2011@163.com (S.-Q.H.); wjie68@163.com (J.W.); 2 Chengdu Academy of Agricultural and Forestry Sciences, Chengdu 611130, China; yangxue790702@163.com

**Keywords:** rabbit, chronic heat stress, transcriptome, differentially expressed genes

## Abstract

**Simple Summary:**

It has been widely acknowledged in farm animals that environmental heat stress would have comprehensive influences on many kinds of physiological aspects, including the metabolic characteristics, production performances, welfare concerns, etc. The rabbit is a small herbivore and needs to regulate the body temperature in a fine mechanism. Little is known, however, about the genes and pathways that are involved in the regulatory responses under chronic heat stress conditions. In the present study, we investigated the liver transcriptome changes in response to chronic heat stress for Hyla rabbit, that is a commercial meat breed recently introduced into China. We successfully revealed the differentially expressed genes that were significantly enriched in heat stress related biological processes. The results would help us for better understanding the molecular mechanisms underlying physiological responses against heat stress in rabbits.

**Abstract:**

Rabbit is an economically important farm animal in China and also is a widely used animal model in biological researches. Rabbits are very sensitive to the environmental conditions, therefore we investigated the liver transcriptome changes in response to chronic heat stress in the present study. Six Hyla rabbits were randomly divided into two groups: chronic heat stress (HS) and controls without heat stress (CN). Six RNA-Seq libraries totally yielded 380 million clean reads after the quality filtering. Approximately 85.07% of reads were mapped to the reference genome. After assembling transcripts and quantifying gene expression levels, we detected 51 differentially expressed genes (DEGs) between HS and CN groups with thresholds of the adjusted *p*-value < 0.05 and |log2(FoldChange)| > 1. Among them, 33 and 18 genes were upregulated and downregulated, respectively. Gene ontology analyses further revealed that these DEGs were mainly associated with metabolism of lipids, thyroid hormone metabolic process, and cellular modified amino acid catabolic process. The upregulated *ACACB*, *ACLY*, *LSS*, and *CYP7A1* genes were found to be inter-related through biological processes of thioester biosynthetic process, acyl-CoA biosynthetic process, acetyl-CoA metabolic process, and others. Six DEGs were further validated by quantitative real-time PCR analysis. The results revealed the candidate genes and biological processes that will potentially be considered as important regulatory factors involved in the heat stress response in rabbits.

## 1. Introduction

Global warming has become a serious environmental factor for humans and livestock. Heat stress due to global warming results in a variety of animal production parameters, impaired metabolism, and even death in extreme cases. Environmental heat stress negatively impacts animals, resulting in significant welfare concerns and economic losses in livestock industries. Long-term high temperatures and excessively warm weather generate serious heat stress for animals, which can cause inflammatory response [1], improve immune status, and alter hepatic gene expression [2] in the dairy cow. Study on broiler liver transcriptome reveals that treatment with cyclic high ambient temperature caused metabolic, physiologic, and cellular-level changes [3]. Effects of heat stress on rabbits have been widely documented to include collapse inefficient thermoregulatory mechanism, compromised the immune system, and decline in the antioxidant defense system [4].

RNA-Seq has been widely used in recent studies to investigate differentially expressed genes (DEGs) related to heat stress. For example, Jastrebski et al. [5] found genes related to cell cycle regulation, DNA replication, and DNA repair along with immune function were changes when chicken under chronic heat stress condition. Kim et al. [6] found *PIK3R6*, *PIK3R5*, and *PIK3C2B* have an important relationship with the mechanisms of adaptation to heat stress in ducks. Using transcriptome analysis of liver tissue, Li et al. [7] found the pathways of carbon metabolism, the PPAR signaling pathway, and vitamin digestion and absorption are heat stress related and, *APOA4* and *APOA5* might function synergistically to regulate the anti-heat stress ability in Hu sheep. Similarly, Lu et al. [8] found DEGs under heat stress condition were significantly associated with biological processes such as response to stress, immunoreaction, and fat metabolism.

Despite the above-mentioned publications, studies investigating DEGs and pathways related to heat stress in rabbit are rare. Rabbits have several unique metabolic features that are similar to humans, so they provide a unique system and are widely used for the study of liver function, gene-targeting and translational research [9]. The liver plays a vital role in the maintenance of body homeostasis and is essential for the coordination of normal metabolism of carbohydrates, lipids, proteins, and vitamins, as well as for biochemical defense against toxic chemicals [10]. Moreover, the liver is more susceptible to oxidative stress than other organs under heat stress and, thus, was an ideal candidate tissue to study the impact of that stress on organismal energy transformation, hormone metabolism and immune response. However, the molecular underpinnings of gene regulation to heat stress are poorly understood on liver transcriptome. Our results provide insights into the molecular mechanisms associated with the liver’s response to chronic heat stress. The comprehensive examination of the genes and regulatory pathways related to rabbit chronic heat stress response will assist in the ultimate goal of breeding practices that will help to deal with high ambient temperatures.

## 2. Materials and Methods

### 2.1. Ethical Statements

This study was carried out at the experimental farm of Sichuan Agricultural University in Ya’an, China, from September to October 2018. All procedures involved in the present study were approved by Institutional Animal Care and Use Committee of Sichuan Agricultural University (DKY-B20171906).

### 2.2. Experiment Design and Sample Collection

The study was conducted in Hyla rabbits. After weaning at 30 days of age, animals were routinely fed the commercial pelleted food, for which ingredients and chemical composition are shown in Table S1. At the 70 days of age, six healthy and comparative female Hyla rabbits were randomly recruited with body weight of 2079.2 ± 29.6 g. All animals had been maintained in an air-conditioned room for a pretreatment period of one week at ~21.5 °C. After which, all rabbits were individually housed in stainless steel cages where diet and water were offered *ad libitum*, and randomly divided into two groups (*N* = 3 for each group). During the entire experimental period, ambient temperatures and relative humidity were daily recorded at 08:00, 12:00, and 18:00, respectively. The ambient temperature and humidity were measured by a temperature-humidity detector (Elitech, Beijing, China). The control group (CN) was housed in an air-conditioned room with a temperature-humidity index (THI) of 24.13 ± 1.70 and treated as the absence of heat stress. The treatment group (HS) were similarly housed in an air-conditioned room with a THI of 29.89 ± 0.96, which is higher than the recommended threshold (27.8) of THI index under heat stress condition [11]. The formula of THI index is:

THI = t − [(0.31 − 0.31 RH)(t − 14.4)](1)

where RH = relative humidity/100 and t = ambient temperature.

During the pretreatment and experimental periods, the clinical signs of respiration rate, heart rate, and rectal temperature of all animals were recorded for evaluating the degree of response to heat stress. Data among groups were statistically analyzed with a one-way ANOVA test of the SPSS v11.0 software. The significant differences among means were compared using Duncan’s multiple-range test. All data are expressed as least squares means ± standard errors (mean ± SE). All of these individuals were stunned by electro-anesthesia and sacrificed by jugulation at the end of experimental period, the tissue used is the left lobe of liver and selected within a relatively homogenous portion of which was free of vasculature, and then immediately frozen in −80 °C for RNA-seq analysis.

### 2.3. RNA Extraction, cDNA Library Construction and Sequencing

Total RNA was isolated from the six liver samples using TRIzol Reagent (TaKaRa, Dalian, China), according to the standard protocol, for which the DNA was cleaned out using DNaseI. RNA concentration, purity, and integrity were measured using the NanoPhotometer^®^ spectrophotometer (IMPLEN, Westlake Village, CA, USA) followed by RNA Nano 6000 Assay Kit on the Bioanalyzer 2100 system (Agilent Technologies, Palo Alto, CA, USA). RNA quality was verified by ensuring all RNA samples had an absorbance (A260/280) of between 1.8 and 2, and RNA integrity number of between 7.5 and 10 were deemed to be of sufficiently high quality.

Sequencing libraries were generated using the NEBNext^®^ Ultra^TM^ RNA Library Prep Kit for Illumina^®^ (NEB, Beverly, MA, USA) following manufacturer’s recommendations. Briefly, mRNA was purified from total RNA using poly-T oligo-attached magnetic beads and fragmented by an Illumina proprietary fragmentation buffer with an elevated temperature. Random hexamer primer and M-MuLV Reverse Transcriptase (RNase H^−^) were used to synthesizes first strand cDNA. Second stand cDNA synthesis was subsequently performed using DNA polymerase I and RNase H. End repair, A-tailing, adaptor ligation, and cDNA purification and enrichment were then performed. Sequencing was performed using an Illumina HiSeq 2500 platform and 150 bp paired-end reads were generated.

### 2.4. Quality Control and Mapping of Reads

The raw reads were generated from Illumina sequencing machine in FASTQ format [12]. All of them were subjected to quality control to remove adaptor sequences and low-quality reads. In this step, The Phred score Q20, Q30, and GC content of the raw reads were calculated. After removing these reads containing adaptors, >10% of ambiguous ‘N’ bases and with low quality (>50% of bases with Phred scores ≤ 20), the remaining clean reads for each sample were aligned to rabbit reference genome (*OryCun*2.0.75 in Ensembl) using HISAT2 v2.0.5 software [13].

### 2.5. Analysis of Differentially Expressed Genes

We used featureCounts v1.5.0-p3 [14] to count the number of mapped reads to each gene, which were normalized for the gene length and library size. And the gene expression was calculated in fragments per kilo-base of exon per million mapped fragments (FPKM). Which considers the effect of sequencing depth and gene length for the reads count, is used for estimating gene expression levels. Pearson’s correlation coefficients (R^2^) of 2 individuals in the same group were checked on the basis of the FPKM value of each sample to reflect the accuracy and reliability of the results; the closer R^2^ is to 1, the higher the similarity of the expression pattern between samples. Generally, R^2^ > 0.8 was considered suitable. For exploring gene expression profile in each sample, all the genes were categorized into five groups based on their FPKM values: very low expression gene (0–1), low expression gene (1–3), medium expression gene (3–15), high expression gene (15–60), and extremely high expression gene (>60).

The DEGs were analyzed using DESeq2 v1.16.1 software [15] between HS and CN groups. In which, the DEGs were selected by thresholds of |log2(FoldChange)| > 1 and pad < 0.05. Clustering analysis of the detected DEGs was subsequently carried out using the heatmaps in R software package.

### 2.6. Functional Enrichment Analysis

Gene ontology (GO) is an international standard gene functional classification system that describes three ontologies: molecular function, cellular component, and biological process. To evaluate the relevance of DEGs, and effects on signaling pathways at chronic heat stress, we performed GO analysis for biological processes with clusterProfiler R package as described with Yu et al. [16] with a Benjamini-Hochberg adjusted *p*-value of <0.05.

ClueGO integrates GO terms as well as KEGG/BioCarta pathways and creates a functionally organized GO/pathway term network, which can compare clusters of genes and visualizes their functional differences. The ClueGO plug-in v3.7.2 of Cytoscape was used to visualize non-redundant biological terms for genes in functionally grouped networks [17].

### 2.7. Validation of RNA-Seq Data by qPCR

To confirm the repeatability and reproducibility of DEGs obtained from RNA-Seq, 6 DEGs were chosen randomly for qPCR validation. The primers for the qPCR were designed using Primer Premier 5.0 software based on consensus cDNA sequence of each gene downloaded from the NCBI database, and the primer sequences are listed in Table S2. The liver tissues were used for RNA extraction by RNAiso Pure RNA Isolation Kit (TaKaRa, Dalian, China). Single stranded cDNA was synthesized from 1.5 μg of RNA using a PrimeScript RT reagent kit (TaKaRa, Dalian, China). qPCR was performed on Bio-Rad CFX96 real-time PCR detection system (Bio-Rad, Inc., Hercules, CA, USA). PCR reaction was done in a final volume of 10 μL/well including 5 μL SYBR Green Super Mix (Bio-Rad, Hercules, CA, USA), 1 μL template cDNA, 0.4 μL of each primer (10 pmol/μL), and 3.2 μL of double-distilled water. Reaction condition is 95 °C for 3 min, 40 cycles of 95 °C for 5 s, and 30 s at the Tm, subsequently 95.0 °C for 10 s, then Melt Curve from 65 °C to 95 °C per increment 0.2 °C for 10 s to read plate. The standard curves were diluted 10-fold gradient from 10^−3^ to 10^−9^ to ensure the amplification efficiency in 100% ± 5%. The 6 samples were run in triplicate for the six genes; each run contains a non-template control. In relative quantify the expression of the DEGs, the qPCR data is presented relative to *GAPDH* referred to as an internal control, and the method of 2^−ΔΔ*C*T^ was enrolled to calculate which using quantification cycle values as described by Livak and Schmittgen [18].

## 3. Results

### 3.1. Influence of Chronic Heat Stress on Physiological Characteristics

Respiration rate, heart rate, and rectal temperature are shown in Table 1. At pretreatment, there were no significant differences in the changes of rabbit respiration rate, heart rate, and rectal temperature among the HS and CN groups. On the other hand, no significant difference parameters were found in the animals of CN group from pretreatment period to experiment period. Which exhibited that CN animals were absence of heat stress during the entire experimental period. After the heat stress treatment, heart rate in the HS group (245 ± 35) was significantly higher than in the CN group (213 ± 29), Similarly, rectal temperature, was also significantly (*p* < 0.01) elevated in the HS group compared with the CN group.

### 3.2. Sequencing and Mapping of Reads

A total of 387,409,896 raw reads were generated from livers of all the six rabbits. After filtering adaptor sequences and low quality reads, the number of clean reads were 379,542,170. The GC content of these six samples were about 55%. All Q20 values of the read sequences in the samples exceeded 95.95%, and Q30 exceeded 90.05%. The results of mapping showed that more than 83.65% of the reads (total mapped reads) matched the reference genome and the remaining were unmatched. Of these, more than 80.66% of the reads were matched to a unique genomic location and less than 3.54% of the reads were matched to multiple genomic locations (Table 2). The reads of unique mapped on the rabbit reference genome were used for further bioinformatic analysis.

### 3.3. Analysis of Gene Expression

We estimated the expression levels of mRNAs through FPKM values. The value of Pearson’s correlation coefficients (R^2^) was 0.960–0.974 among HS1, HS2, and HS3, and 0.946–0.967 among CN1, CN2, and CN3 (Figure S1), which confirmed the sufficiently high similarity between the 3 biological replicates of the two rabbit groups. As shown in Table 3, the overall gene expression levels of the six samples were similar, but small percentage changes of various expression quantities among them. Most gene expressed either very low level or medium level, approximately, 29% of genes had FPKM values of less than 1, and 31% had FPKM values in the range of 3–15. Only approximately 8% had FPKM values of more than 60 (Table 3).

**Figure 1:**
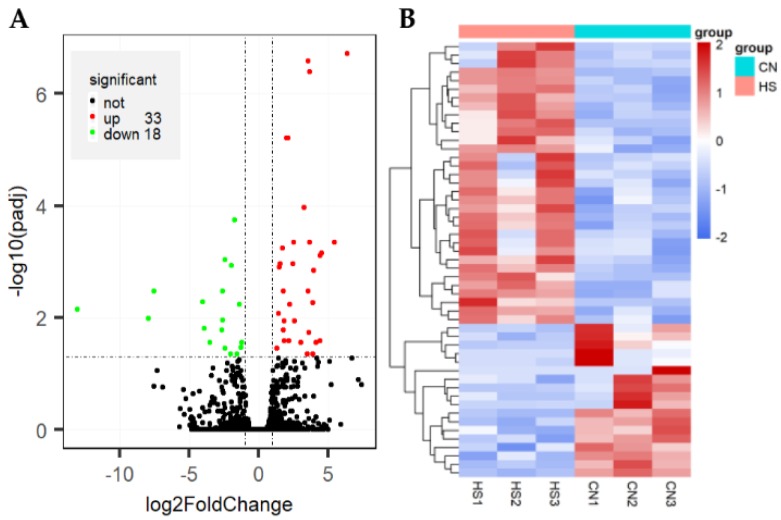
Hyla rabbit genes identified to be differentially expressed between chronic heat stress (HS) and controls without heat stress (CN) animals. (**A**) Volcanic plot of the differentially expressed genes. (**B**) Heat map of the differentially expressed gene cluster analysis.

We compared animals from HS and CN group for differences in gene expression using DESeq2 R package (1.16.1). A total of 51 genes fulfilling both criteria of pad < 0.05 and |log2(FoldChange)| > 1, among them, 33 presented upregulated expression in the rabbit of HS group, while 18 presented downregulated (Table S3, Figure 1A). Hierarchical clustering of the DEGs based on condition (HS vs. CN) was conducted to get a deeper understanding of the gene expression patterns. The results exhibited that the DEGs of rabbit in the HS and CN were clustered into a single class (Figure 1B).

**Figure 2:**
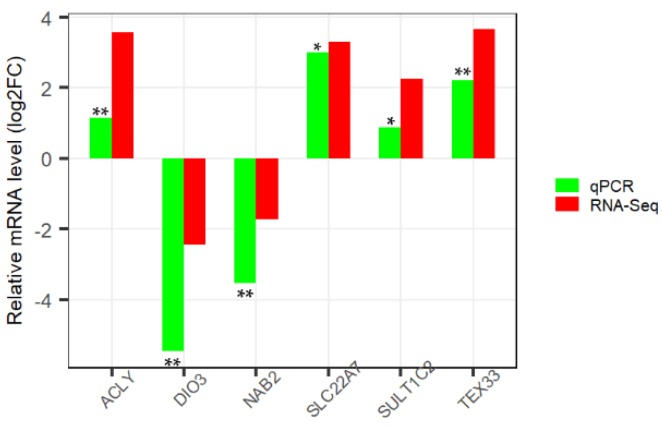
Validation of differentially expressed genes by qPCR.

We detected 6 DEGs using qPCR to verify our RNA-Seq analysis. The expression levels of these genes were analyzed between control and heat stressed samples. The results showed that the tendency of gene expression was concordant with the RNA-Seq result, though the absolute fold changes differed between qPCR and RNA-Seq (Figure 2), thus suggesting the transcriptome sequencing results were reliable.

### 3.4. Functional Categories of DEGs

**Figure 3:**
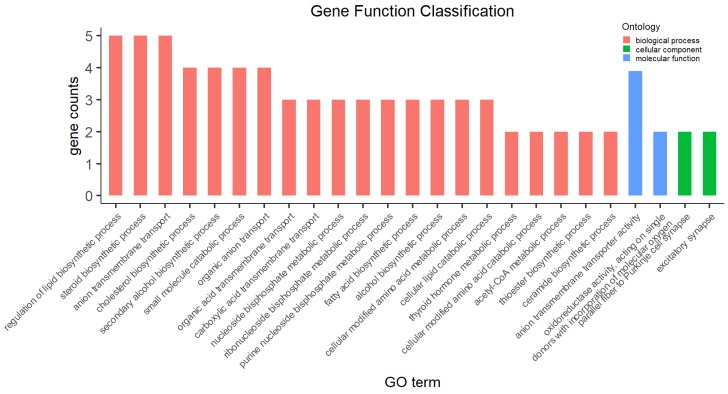
Gene Ontology (GO) analysis enriched by the differentially expressed genes.

Of the 51 DEGs identified between HS and CN rabbits, we found 34 GO terms with pad < 0.05 (Table S4), most of which were associated with a biological process, including metabolism of lipids, thyroid hormone metabolic process, cellular modified amino acid catabolic process, and acetyl-CoA metabolic process (Figure 3). Maximum number of DEGs was enriched for regulation of lipid biosynthetic process, steroid biosynthetic process, and anion transmembrane transport.

**Figure 4:**
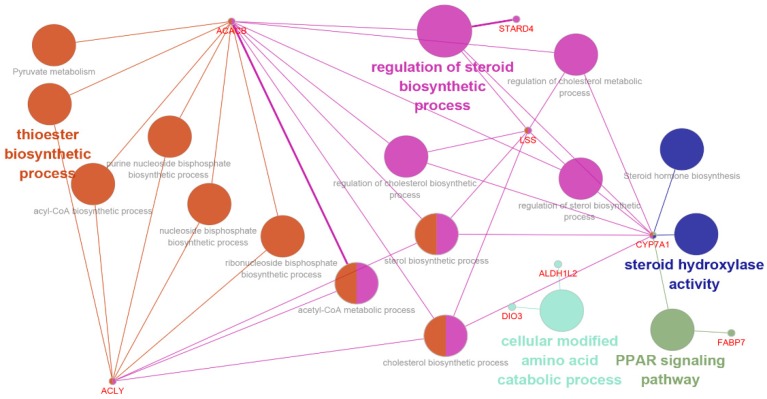
Enrichment analysis of the *ACACB*, *ACLY*, *LSS*, and *CYP7A1* genes using the ClueGO plug-in of the Cytoscape program. The nodes are the GO terms, and edges connect the genes involved in the terms.

Using the ClueGO plug-in, the differentially expressed *ACACB*, *ACLY*, *LSS*, and *CYP7A1* genes were found to be inter-related through biological process of thioester biosynthetic process, acyl-CoA biosynthetic process, acetyl-CoA metabolic process, and other lipid biosynthetic related processes (Figure 4). Interestingly, all of these genes were up-regulated in the HS group.

## 4. Discussion

Heat stress could lead to enormous economic loss in rabbit industry by decreasing feed intake and increasing mortality rate [19,20]. Transcriptome studies have been widely used to explore the underlying molecular basis of heat resistance in animals, such as the cow [2], sheep [8], chicken [3], and duck [6]. In the current study, we artificially induced heat stress condition in rabbit, by which the global gene expression profiles have been studied. Our results revealed the important candidate genes and pathways in relation with heat stress in rabbit.

The accumulating evidence indicated heat stress has a severe effect on the health of animals and cause endocrine changes such as in measurable levels of circulating cytokines and corticosteroids [21], and gene expression including specific and highly regulated signaling cascades leading to the transcriptional regulation of endogenous antioxidant enzymes [22]. Through transcriptomic analysis of broilers under high ambient temperature, Coble et al. [3] found the DEGs were mainly associated with cell signaling and endocrine system development and function. A study on cows under heat stress found the increased basal and stimulated insulin levels, which resulting in decreased adipose tissue lipid mobilization and apparently increased glucose utilization by peripheral tissues [23]. In this study, we found the associated DEGs were enriched in fatty acid biosynthetic process, acetyl-CoA metabolic process, and acyl-CoA biosynthetic process, which showed the influence on fat metabolism under heat stress, and was similar with the result of liver transcriptome analysis in sheep [7]. Furthermore, the differentially expressed *ACACB*, *ACLY*, *LSS*, and *CYP7A1* genes were found to be inter-related through biological processes of lipid biosynthetic related processes. The acetyl-CoA carboxylase beta (*ACACB*), converts acetyl-CoA to malonyl-CoA, which inhibits carnitine palmitoyl-CoA transferase I, the rate-limiting step in fatty acid uptake and oxidation by mitochondria [24]. *ACACB* may be involved in the regulation of fatty acid oxidation and associated with metabolic syndrome and diabetes [25]. ATP citrate lyase (*ACLY*), a cytosolic enzyme that catalyzes the generation of acetyl-CoA for both fatty acid and cholesterol synthesis is involved in do novo lipogenesis pathway [26]. And heat stress result in hepatic expression of key lipogenic proteins and increased *ACLY* expression in chicken [27]. The lanosterol synthase (*LSS*) is a well-known enzyme within the pathway of cholesterol biosynthesis, which catalyzes the reaction of conversion from 2,3-oxide-squalene to lanosterol [28]. And the cholesterol-7-alpha hydroxylase (*CYP7A1*), is the rate-limiting enzyme involved in the biosynthesis of bile acid from cholesterol and participate in the degradation of cholesterol in the liver [29,30]. *CYP7A1* has an important role in cholesterol metabolism, changes in hepatic *CYP7A1* mRNA expression are correlated with serum corticosterone levels [31]. Importantly, all of these genes were up-regulated in heat stressed Hyla rabbit. Therefore, these four genes could potentially be considered as important novel regulatory factors involved in the heat stress response in Hyla rabbit.

Heat stress response is very complex process and induce changes in gene expression transcript profiles, including those related to fatty acid synthase activity, oxidoreductase activity, and lipid peroxidation [32]. Acute and chronic heat stresses exhibit different responses on production and metabolism. Stress challenges the homeostatic state of the organism. Thus, the stress response includes complex responses to maintain a steady state. Study in chicken found acute heat stress cause a stronger response than chronic ones, and there are more DEGs [33]. Previous studies reported that fatty degeneration with dilation of sinusoid, and necrosis with heterophils and lymphocytes was observed [34], but did not induce oxidative damage [33,34,35] in chronic heat stress, and we found similar results in rabbit liver of the responses involved biological process of thioester biosynthetic process, acyl-CoA biosynthetic process, acetyl-CoA metabolic process, and other lipid biosynthetic related processes, and out of oxidative damage related pathways. The purpose of the responses may be to alleviate the effects of heat stress.

## 5. Conclusions

Liver transcriptome response to chronic heat stress have been examined using RNA-Seq technology in Hyla rabbit. A total of 51 DEGs were screened out, which represents the genes involved in thermoregulation mechanism and acclimation. Furthermore, the upregulated *ACACB*, *ACLY*, *LSS*, and *CYP7A1* genes were found to be inter-related through heat stress related biological processes. The data obtained represented a resource for further investigations of the function of these four candidate genes, and our findings revealed these genes and biological processes that will potentially be considered as important regulatory factors involved in the heat stress response in rabbits.

## Figures and Tables

**Table 1:** Physiological characteristics of animals.

Item	Pretreatment		Treatment	
	HS	CN	HS	CN
respiration rate (/min)	120 ± 11	126 ± 17	126 ± 14	124 ± 17
heart rate (/min)	210 ± 27 ^B^	223 ± 30 ^AB^	245 ± 35 ^A^	213 ± 29 ^B^
rectal temperature (°C)	39.5 ± 0.3 ^B^	39.6 ± 0.2 ^B^	40.6 ± 0.4 ^A^	39.5 ± 0.3 ^B^

In the same row, ^A,B^ letters mean significant difference at 0.01 levels.

**Table 2:** Mapping results of the transcriptome data.

Items	HS1	HS2	HS3	CN1	CN2	CN3
Raw reads	64,568,316	64,568,316	64,568,316	64,568,316	64,568,316	64,568,316
Clean reads	63,191,812	63,262,266	63,292,936	63,065,116	63,170,720	63,559,320
Clean bases	9.48G	9.49G	9.49G	9.46G	9.48G	9.53G
Error rate	0.03%	0.03%	0.03%	0.03%	0.03%	0.03%
Q20	96.50%	95.95%	96.52%	96.38%	97.38%	97.70%
Q30	91.13%	90.05%	91.09%	90.82%	92.87%	93.58%
GC content	55.51%	55.76%	54.95%	55.30%	55.83%	55.52%
Total map	53,694,163 (84.97%)	52,919,164 (83.65%)	54,192,938 (85.62%)	53,806,908 (85.32%)	53,743,538 (85.08%)	54,514,687 (85.77%)
Unique map	51,618,791 (81.69%)	51,024,408 (80.66%)	51,954,752 (82.09%)	51,853,433 (82.22%)	51,701,154 (81.84%)	52,454,172 (82.53%)
Multi map	2,075,372 (3.28%)	1,894,756 (3.0%)	2,238,186 (3.54%)	1,953,475 (3.1%)	2,042,384 (3.23%)	2,060,515 (3.24%)
Proper map	48,422,878 (76.63%)	47,632,360 (75.29%)	48,881,796 (77.23%)	48,638,926 (77.12%)	48,810,104 (77.27%)	49,723,966 (78.23%)

HS1, HS2, and HS3 were animals in the HS (chronic heat stress) group and CN1, CN2, and CN3 were animals in the CN (controls without heat stress) group.

**Table 3:** Number of genes at different expression levels.

FPKM Interval	HS1	HS2	HS3	CN1	CN2	CN3
0–1	4319 (28.65%)	4315 (28.56%)	4373 (28.79%)	4349 (29.08%)	4497 (29.08%)	4582 (29.47%)
1–3	2379 (15.78%)	2413 (15.97%)	2399 (15.79%)	2315 (15.48%)	2426 (15.69%)	2397 (15.42%)
3–15	4657 (30.89%)	4635 (30.68%)	4748 (31.26%)	4523 (30.24%)	4829 (31.23%)	4916 (31.62%)
15–60	2448 (16.24%)	2492 (16.49%)	2411 (15.87%)	2468 (16.50%)	2545 (16.46%)	2542 (16.35%)
>60	1272 (8.44%)	1254 (8.30%)	1259 (8.29%)	1300 (8.69%)	1167 (7.55%)	1110 (7.14%)
Total	15,075	15,109	15,190	14,955	15,464	15,547

FPKM means fragments per kilobase of transcriptome per million mapped reads, indicating the gene expression level.

## Supplementary Materials

The following are available online at https://www.mdpi.com/2076-2615/9/12/1141/s1. Figure S1: The correlation of FPKM values of all genes between the three replicates of HS and CN group, Table S1: Ingredients and chemical component of the experimental diet, Table S2: Primers used in this study for qPCR, Table S3: List of genes expression that were differentially expressed between HS and CN animals, Table S4: GO analysis of differentially expressed genes in heat stressed and control rabbit groups.
