# Supplementary material for: Liver Transcriptome Changes of Hyla Rabbit in Response to Chronic Heat Stress
Source: Animals (Basel). 2019 Dec 13;9(12):1141. doi: 10.3390/ani9121141 (PMC6940982; doi:10.3390/ani9121141)
Supplement: Supplementary file 1 [file animals-09-01141-s001.zip › Supplementary_files/Table S1.docx]

**Table S1.** **Ingredients and chemical component of the experimental diet.**

| **Ingredient** |  | **Diet (%)** |
| --- | --- | --- |
| alfalfa grass |  | 30.00 |
| Barley grain |  | 23.50 |
| corn |  | 8.50 |
| wheat bran |  | 10.00 |
| soya bean meal |  | 23.50 |
| Molasses |  | 0.35 |
| choline chloride |  | 0.15 |
| Di-calcium phosphate |  | 1.65 |
| Sodium chloride |  | 0.30 |
| calcium hydrophosphate |  | 0.35 |
| DL-Methionine |  | 0.20 |
| Meat rabbit premix |  | 1.50 |
| Total |  | 100 |
|  |  |  |
| **Chemical Component** |  |  |
| Dry matter (DM) |  | 85.83 |
| Crude protein (CP) |  | 16 |
| Crude fiber (CF) |  | 14.25 |
| Ether extract (EE) |  | 3.51 |
| Metabolizable energy (ME, MJ/Kg) |  | 10.8MJ/kg |
| Calcium |  | 0.9 |
| Phosphorus |  | 0.61 |
| Lysine |  | 0.91 |
| Methionine |  | 0.36 |
| Threonine |  | 0.59 |
| Tryptophan |  | 0.26 |
