# Supplementary material for: Liver Transcriptome Changes of Hyla Rabbit in Response to Chronic Heat Stress
Source: Animals (Basel). 2019 Dec 13;9(12):1141. doi: 10.3390/ani9121141 (PMC6940982; doi:10.3390/ani9121141)
Supplement: Supplementary file 1 [file animals-09-01141-s001.zip › Supplementary_files/Table S2.docx]

**Table S2.** **Primers used in this study for qPCR.**

| Gene | Primer sequences (5'-3') | Locus | Tm (°C) | Amplicon size (bp) |
| --- | --- | --- | --- | --- |
| *TEX33* | CATTCGCCACAAGTTCG | 4: 84,939,345-84,952,016 | 59.0 | 176 |
|  | TGTAGCCCAGATCGTAGT |  |  |  |
| *SLC22A7* | GAGTGGCTGGATGTGGA | 12: 32,747,199-32,752,781 | 56.3 | 144 |
|  | GGCTAAAGGCAGGGTGA |  |  |  |
| *NAB2* | TGCCTGCCGTGCCTGTCT | GL018714: 251,332-256,179 | 56.3 | 138 |
|  | GGCTCTTGGGGCTGAAACT |  |  |  |
| *DIO3* | GACGGCTACCAGGTCTCGG | GL018751: 1,794,253-1,795,100 | 63.0 | 96 |
|  | TCCCTCGGTCGCTTACAC |  |  |  |
| *SULT1C2* | CTTGGGGTTCCTGGTTTGA | 2: 84,304,017-84,324,910 | 63.0 | 103 |
|  | CACTTTGGGTCCTTCTTGATGT |  |  |  |
| *ACLY* | TCAAACGGCGAGGAAAGC | 19: 42,758,242-42,805,994 | 62.3 | 222 |
|  | CGTGGTGGAACAGGACATAGTC |  |  |  |
| *GAPDH* | GAGCACCAGAGGAGGACGA | 9: 10,337,709-10,338,971 | 63.0 | 103 |
|  | TGGGATGGAAACTGTGAAGAG |  |  |  |

*TEX33*: testis expressed 33; *SLC22A7*:solute carrier family 22 member 7; *NAB2*: NGFI-A binding protein 2; *DIO3*: iodothyronine deiodinase 3; *ARG2*: arginase 2; *SULT1C2*: sulfotransferase family, cytosolic, 1C, member 2; *HMGCR*: 3-hydroxy-3-methylglutaryl-CoA reductase; *ACLY*: ATP citrate lyase; *GAPDH:* glyceraldehyde-3-phosphate dehydrogenase.
