# Supplementary figures and images for: Liver Transcriptome Changes of Hyla Rabbit in Response to Chronic Heat Stress
Source: Animals (Basel). 2019 Dec 13;9(12):1141. doi: 10.3390/ani9121141 (PMC6940982; doi:10.3390/ani9121141)

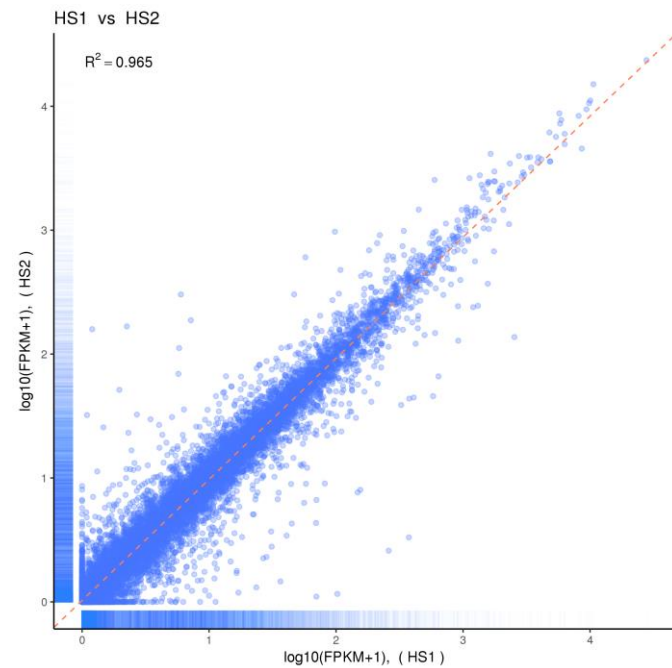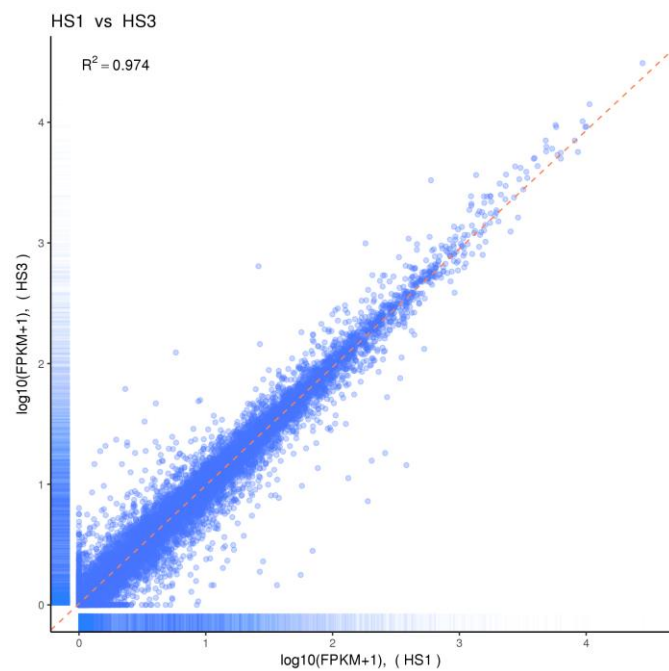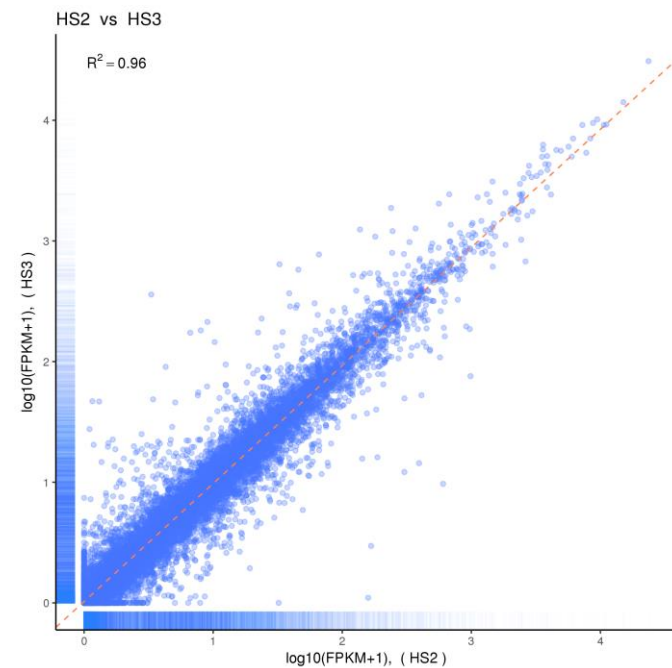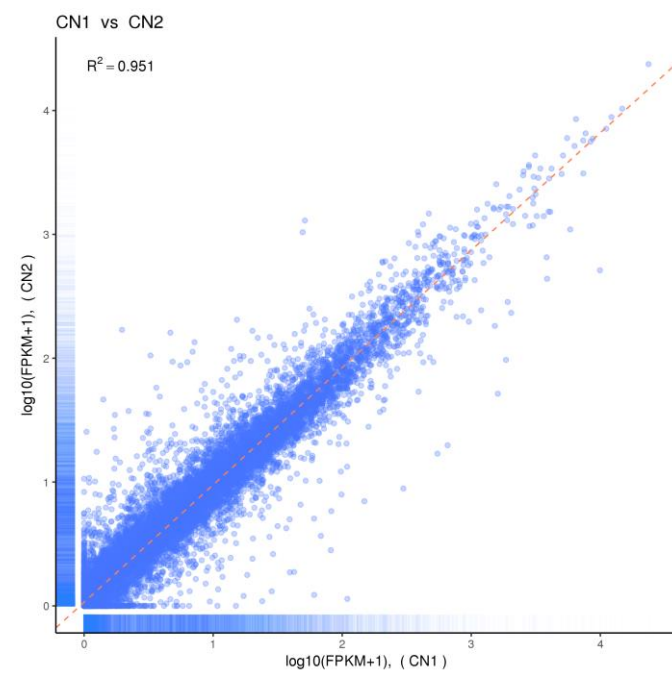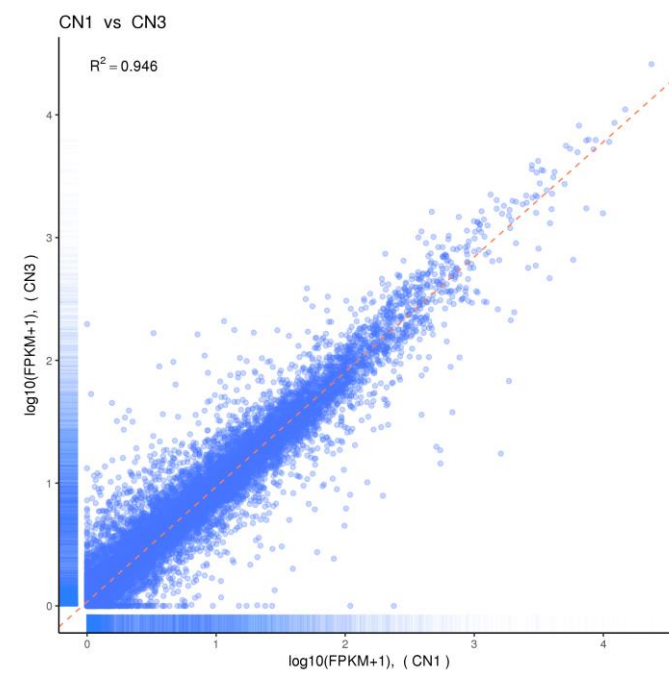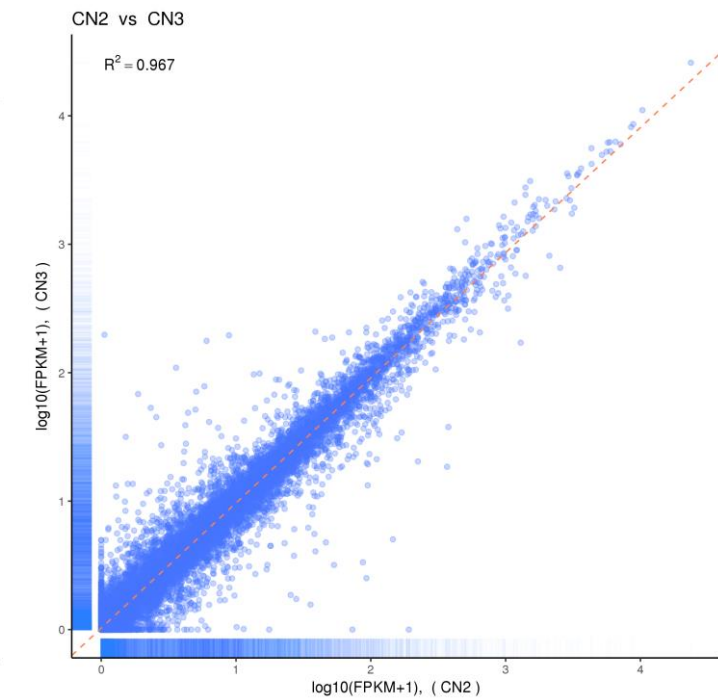

Supplement: Supplementary file 1 [file animals-09-01141-s001.zip › Supplementary_files/Figure S1. The correlation of FPKM values of all genes between the three replicates of HS and CN group.pdf]
